# Oculogyric Crisis Due to Aripiprazole Ingestion as a Suicide Attempt: A Case Report

**DOI:** 10.7759/cureus.48267

**Published:** 2023-11-04

**Authors:** Ammar Bafarat, Badr Alaseeri, Suhail A Labban, Roaa E Morya

**Affiliations:** 1 Psychiatry, Ministry of National Guard Health Affairs, Al-Ahsa, SAU; 2 Psychiatry, King Abdulaziz Hospital, Jeddah, SAU; 3 Psychiatry, Ministry of National Guard Health Affairs, Jeddah, SAU; 4 Medicine, College of Medicine, King Saud Bin Abdulaziz University for Health Sciences, Jeddah, SAU

**Keywords:** oculogyric crisis, extrapyramidal side effects, antipsychotic, depression, aripiprazole, suicide attempt, acute dystonia

## Abstract

Oculogyric crisis (OGC) is a rare type of acute dystonia characterized by spasmodic upward deviation of the eyes lasting for a few minutes to several hours. It is commonly seen with the administration of first-generation antipsychotics and rarely reported in patients taking second-generation antipsychotics. Although aripiprazole, a second-generation antipsychotic, is known for its low potential for extrapyramidal side effects (EPS), there are multiple case reports of it resulting in acute dystonia, especially OGC. In this paper, we report a case of aripiprazole-induced OGC in a 16-year-old female patient after a suicide attempt by taking 40 mg of aripiprazole and 5 g of acetaminophen. The necessary investigations were ordered, and the patient’s dystonic symptoms resolved completely after administering parenteral diazepam and benztropine.

## Introduction

Aripiprazole is a second-generation antipsychotic medication used to treat many psychiatric conditions. It works as a partial agonist to D2 dopaminergic receptor in the central nervous system, eliciting a similar but reduced effect as dopamine. In addition, it works as a partial agonist on 5HT1A receptor, but as an antagonist in 5HT2A receptor. Due to its unique mechanism of action, aripiprazole is known to cause less extrapyramidal side effects (EPS) such as acute dystonia, parkinsonism, tardive dyskinesia, and akathisia. Thus, it is preferred in children and adolescents [1,2]. The established efficacious and tolerable dose of aripiprazole is within the dose range of 10-30 mg/day for schizophrenia and 15-30 mg/day for manic or mixed states associated with bipolar I disorder [3].

Acute dystonia is described as sudden involuntary muscle contraction and spasm involving muscles present in the eyes, mouth, chin, and neck. It is usually reported to be caused by first-generation antipsychotics rather than second-generation antipsychotics. A sub-type of acute dystonia is oculogyric crisis (OGC), which is described as aggressive, sustained, and bilateral fixed upward gaze of the eyes [1,4]. The Nova Scotia Early Psychosis Program estimates the incidence of OGC caused by second-generation antipsychotics to be 1.8% [5]. More specifically, a study assessed the clinical effects of isolated aripiprazole exposure. The occurrence of dystonia was estimated to be 1.7% [6]. Risk factors include young age, male gender, drug naivety, up-titration or initiation of a new antipsychotic, and any personal or family history of acute dystonia [4]. As discussed before, aripiprazole is mostly used in children and adolescents because it is less likely to cause EPS [1,2]. Thus, such presentation of OGC is considered rare in the literature.

## Case presentation

A 16-year-old Saudi female patient, known case of major depressive disorder with psychotic features, was brought to the Emergency Department (ED) by her mother as she started to complain of sustained, painful contractions of the neck muscles and fixed up-rolling of the eyes that lasted two hours. A detailed history was taken from the patient and a collateral history was taken from her mother. The history revealed that the patient ingested 10 tablets of paracetamol 500 mg and four tablets of aripiprazole 10 mg 12 hours prior to the presentation as part of a suicide attempt due to high school stress. Her mother added that she immediately vomited. 

There was no family history of psychiatric or medical illnesses. The patient’s parents are divorced, and she lives with her mother. She is in eleventh grade, and her school performance is excellent. She did not report any social withdrawal. She reported smoking one cigarette a day. She does not have any allergies to any food or medications. 

On examination, the patient was conscious, alert, and oriented to time, place, and person. However, she was in distress. She was also experiencing a headache and blurry vision. She could bring her eyes and neck to original position only for a few seconds on command. Her Glasgow Coma Scale (GCS) score was 15/15. Cranial nerves exam was intact. The rest of the physical examination was unremarkable. The patient’s venous blood gas (VBG), urine and electrolytes (U&E), liver function test (LFT), troponin, complete blood count (CBC), and creatine phosphokinase (CPK) values were within the normal range. CT scan of the brain and chest X-ray (CXR) were unremarkable as well.

The patient received diazepam 2 mg intramuscularly (IM) in the ED, and an urgent consultation was sent to a psychiatrist. The psychiatrist assessed the patient and noted old self-harm marks on both wrists. She was admitted as a case of acute dystonia with OGC and torticollis. Management was initiated by administering benztropine 2 mg IM. Toxicological screen to rule out substance use and to document acetaminophen blood level was ordered as well. The patient and her family were educated regarding acute dystonia and other extrapyramidal symptoms that can be caused by antipsychotics.

The spasmodic movements of the eyeballs and neck resolved 30 minutes after the administration of benztropine. The patient reported regretting the ingestion of the medications and denied having any current death wishes, suicidal intentions, plans, or previous attempts. Upon further assessment by the psychiatrist, the patient gave history that she was diagnosed as a case of major depressive disorder with psychotic features two years ago in a private clinic as she was complaining of low mood, loss of interest, sleep disturbances, death wishes, and auditory hallucinations. She described her previous hallucinations as multiple whispering voices that are commentary and non-commanding. She was started on aripiprazole 10 mg tab PO/OD for a few weeks, which did not improve her symptoms or cause any EPS. Thus, she was switched to escitalopram 10 mg tab PO/OD. The patient was well controlled upon escitalopram 10 mg tab PO/OD but stopped it a few months ago. As a result, she started to experience the low mood, auditory hallucinations, and insomnia again. Risk assessment was done using SAD PERSONS scale and scored (3), which indicates low risk. Mental status examination (MSE) did not reveal any current hallucinations or delusions. Upon inquiry about the wrist self-harm marks, the patient reported that she harmed herself six months ago using a blade, and it was a non-suicidal self-injury to alleviate overwhelming negative emotions. 

An admission decision was made by the medical team to observe the patient for 24 hours for acetaminophen toxicity. However, the family signed a Discharge Against Medical Advice (DAMA) form and, thus, acetaminophen level investigation was not done. The patient was given a clinic appointment with the psychiatrist three days after the discharge.

The patient and the mother attended the clinic appointment and escitalopram 10 mg tab PO/OD and mirtazapine 15 mg tab PO/HS were prescribed for her, and a close follow-up appointment was booked for her.

## Discussion

Although acute dystonia with atypical antipsychotics is relatively rare, several case reports have reported various acute dystonic adverse effects after initiating treatment with aripiprazole, most of which were among young, male patients [4,7,8]. Moreover, association with eye pain, blurring of vision, headache, dizziness, anxiety, and difficulty in bringing the eyes to the original position was noted in most cases. However, all the associated symptoms were resolved once OGC was treated with anticholinergics or aripiprazole was discontinued [4,7,8]. Similarly, in this case, the patient experienced a headache and blurring of vision in addition to feeling anxious. All her symptoms were also resolved once OGC was resolved.

As per the acetaminophen ingestion, the patient was not a candidate for oral gastrointestinal decontamination using activated charcoal since she came 12 hours after the ingestion. Additionally, acetaminophen serum level was not obtained since the family signed a DAMA form and took the patient home. N-acytylcystine was also not administered as the acetaminophen dose the patient took does not equal or exceed the minimum toxic single dose, which is 7.5 to 10 g [9]. 

Based on a systematic review discussing the spectrum of disorders associated with OGC, it was observed that OGCs were related to three main categories of disorders: drug-induced disorders, hereditary and sporadic movement disorders, and disorders related to focal brain lesions. The majority of cases were drug-induced and most commonly associated with neuroleptics, antiemetics, or other dopamine antagonists [10]. This article discusses a case of a second-generation antipsychotic-induced OGC, aripiprazole. In this case, OGC was described as an acute dystonic reaction occurring promptly after the ingestion of 40 mg of aripiprazole as part of a suicide attempt. The pathophysiology of developing OGC after antipsychotics remains unclear. However, dystonic symptoms are typically related to functional disruption of dopaminergic neurotransmission [10].

In drug-induced OGCs, the initial step of management is to stop or, if not possible, reduce the dose of the offending agent. Moreover, administration of anticholinergics, such as benztropine, can alleviate acute dystonic symptoms within minutes. If the patient does not respond after 15-30 minutes, another dose should be administered. In case there is a persistent lack of response to anticholinergics, oral benzodiazepines might provide symptom relief. Oral administration of oral anticholinergics for 4-7 days is also recommended to prevent recurrence of symptoms after resolution [10].

## Conclusions

Even though aripiprazole is safer than other antipsychotics in terms of extrapyramidal adverse effects, this possibility should always be taken into consideration as this case highlights a rare dystonic side effect that can be caused by using antipsychotics. Although not fatal, oculogyric crisis causes significant distress and discomfort in patients, which puts them at an increased risk of treatment noncompliance and relapse. Therefore, caution and close monitoring for acute dystonic reactions are needed when initiating aripiprazole or any other antipsychotics.
